# Approach for Hemolymph Collection and Biochemical Profiling of Invasive Callinectes sapidus: Methodology and Physiological Assessment

**DOI:** 10.3390/ani16121894

**Published:** 2026-06-18

**Authors:** Laura Gentile, Maria Giulia Ferrari, Asia Ferretti, Alessio Bonaldo, Francesco Dondi, Antonina De Marco

**Affiliations:** Department of Veterinary Medical Sciences, University of Bologna, Via Tolara di Sopra 50, Ozzano Emilia 40064, BO, Italy; laura.gentile4@unibo.it (L.G.); mariagiulia.ferrari2@unibo.it (M.G.F.); asia.ferretti4@unibo.it (A.F.); alessio.bonaldo@unibo.it (A.B.); f.dondi@unibo.it (F.D.)

**Keywords:** invasive species, Portunidae, coagulation, biochemical analysis, physiological indicators, molt-related variability

## Abstract

The blue crab, *Callinectes sapidus*, is an invasive species in the Mediterranean Sea, where it has gained particular attention since 2023 due to its negative ecological and economic impacts. This study aimed to define a standardized method for hemolymph collection and biochemical analysis in male blue crabs from the northern Adriatic Sea, providing data on their physiological status. Hemolymph was collected following a standardized protocol. Preliminary validation tests showed good recovery, stability and linearity results for biochemical analytes, showing no significant analytical interference due to hemolymph pigmentation. Then, key biochemical variables, including glucose, calcium, magnesium, triglyceride, urea, uric acid, aspartate transaminase, gamma-glutamyl transferase, phosphate, potassium, sodium, chloride and total protein, were analyzed. The results revealed considerable variability in the biochemical profiles of the sampled individuals, consistent with the natural heterogeneity expected in a field-collected, mixed-molt cohort, while overall indicating a generally consistent euryhalinity, a good physiological status. The study underscores the importance of standardized protocols for hemolymph analysis in blue crab, contributing to the understanding of its physiology and invasive success.

## Simple Summary

The invasive blue crab, *Callinectes sapidus*, has rapidly spread across Mediterranean and European waters, disrupting native ecosystems and local fisheries. Despite its growing ecological impact, standardized methods for analyzing its hemolymph are lacking. In this research, hemolymph was collected from male blue crabs, and key biochemical parameters were analyzed to develop a reproducible protocol and evaluate their physiological profile. The findings provide biochemical data for the species and contribute to knowledge of natural physiological variability in field-collected individuals and the functional traits driving its invasive success, supporting future health monitoring and ecological risk assessment.

## 1. Introduction

The blue crab (*Callinectes sapidus* Rathbun, 1896) is a portunid crab native to the western Atlantic, ranging from Nova Scotia to Argentina, that has become one of the most successful invasive species in the eastern Atlantic and the Mediterranean Basin [1,2]. Its expansion, facilitated by ballast water transport and aquaculture activities, now includes the Adriatic, North and Baltic Seas, as well as other regions such as Hawaii and Japan. The invasion of *C. sapidus* has drawn significant ecological, economic, and scientific attention, as its omnivorous diet, high fecundity, and tolerance to a wide range of environmental conditions allow it to outcompete native species and disrupt local fisheries [3,4,5].

The remarkable adaptability of *C. sapidus* is closely related to its capacity for osmoregulation and metabolic adjustment to varying salinity and temperature regimes. Hemolymph, the primary circulatory fluid in crustaceans, plays a crucial role in these processes, functioning in the transport of gases, nutrients, metabolites, and ions, as well as in immune defense and homeostatic regulation. Its biochemical composition provides an integrative reflection of the animal’s physiological condition and can serve as a sensitive indicator of stress, molt stage, nutrition, or disease [6,7].

Despite the growing ecological relevance of *C. sapidus* in Mediterranean and European waters, biochemical data on its hemolymph remain limited, and inconsistencies in collection and processing methods can influence analytical results. Several studies describe approaches for collecting crustacean/crab hemolymph and performing biochemical analyses. However, no single universal protocol has yet been adopted. Examining the literature published over the last five years alone, it becomes immediately apparent that a wide range of methodologies coexist, often with a lack of methodological detail. For instance, Wilson et al. (2021) [8] extracted hemolymph from the green shore crab *Carcinus maenas* using a 1 mL syringe and an 18G needle, inserted into the infrabranchial sinus via the arthrodial membrane at the base of the third walking leg. In contrast, Guo et al. (2022) [9] drew hemolymph from the pericardial cavity of the swimming crab *Portunus trituberculatus* using a 2 mL syringe, then allowed the sample to stand overnight at 4 °C to remove gelatinous material, without providing any methodological justification for the subsequent biochemical indices evaluated, including total protein, triglyceride, and glucose. Similarly, Xu et al. (2023) [10], working on *P. trituberculatus*, provided no information whatsoever regarding the collection procedure. Inconsistencies are equally evident when examining studies conducted on the same species within the same year. In 2024, Smalls et al. [11] collected hemolymph from *Callinectes sapidus* through the intersegmental membrane between the posterior carapace and the abdomen using a sterile 1 mL syringe with an 18G needle, whereas Herrera et al. [12] collected hemolymph between the sternites of the first and second pereonites using a 50 µL syringe. Go ahead for the same species, in 2025, Esposito et al. [13] collected hemolymph from *C. sapidus* using a 1 mL sterile syringe with a 26G needle inserted at the leg joints or between the carapace and the abdomen, while Gálvez & Guardiola [14] collected it from the pericardial region via the fifth pereiopod using a 5 mL syringe with a 23G needle. As recently as 2026, Fabrello et al. [15] collected hemolymph from the unsclerotised membrane at the base of the walking legs using a 1 mL plastic syringe, while Freitas-Silva et al. [16] collected it through the thigh junction of the fifth pair of pereopods across three Callinectes species (*C. danae, C. exasperatus*, and *C. marginatus*). This overview illustrates that hemolymph can be drawn from a variety of membranes and body cavities, and Palillo et al. (2021) [17] explicitly noted that this methodological heterogeneity compromises the comparability of results across studies, representing one of the most significant yet underconsidered sources of inconsistency in the field. Discrepancies in measurements extend beyond the collection site to the biochemical analysis itself. For instance, in the study by Simões et al. (2022) [18], hemolymph from blue crabs (*C. sapidus*) was collected post-euthanasia from the base of the chelipeds using an insulin-type syringe and subsequently glucose concentration was then determined using enzymatic colorimetric methods with a specific commercial kit (Labtest Diagnóstica SA). In contrast, in the same year, Hudson et al. [19] collected hemolymph from the branchial cavity of *C. sapidus* of comparable size using a 20-gauge needle, and glucose levels were measured with an Invitrogen™ Amplex Red Glucose Assay kit. Notably, the control crabs in the first study exhibited glucose concentrations around 30 mg/dL, whereas in the second study, levels were approximately 100 µM (≈1.8 mg/dL). These contrasting findings highlight how differences in hemolymph collection sites and subsequent processing can lead to markedly divergent results, contributing to conflicting measurements across studies.

Taken together, the lack of standardization in collection site, processing conditions and analytical procedures represent a fundamental reason why hemolymph studies in arthropods so frequently yield conflicting results. Establishing standardized procedures for hemolymph sampling and serum preparation is therefore essential for obtaining reliable and comparable physiological data. The present study addresses this issue by describing a reproducible method for hemolymph collection and biochemical analysis in male *C. sapidus* specimens from the northern Adriatic coast, which could also be applied to female individuals and other similar crab species. However, when applying this method to females, it is important to account for sex-related variability and reproductive status (ovigerous vs. non-ovigerous) during data analysis, as these factors can significantly influence physiological markers. Adult females possess, for example, sex-specific serum lipoproteins linked to vitellogenesis that markedly alter hemolymph protein, copper and glucose concentrations during ovarian maturation [20,21,22]. To minimize this documented source of variability, the present study was restricted to male specimens. The observed minimum and maximum values of key electrolytes and biochemical constituents are here reported for male specimens, providing preliminary biochemical data to support future studies on the physiological variability and health assessment in this invasive species.

## 2. Materials and Methods

### 2.1. Animals

A batch of fifty blue crabs (*C. sapidus*) was caught at a fishing house in Cesenatico (Adriatic Sea, Italy) (44°12′28″ N, 12°24′11″ E), between September and November 2024. At each sampling event, water temperature, salinity and dissolved oxygen were recorded in situ using multiparametric probe (YSI Pro1020, YSI Incorporated, Yellow Springs, OH, USA). After the immediate sex identification, thirty-eight male crabs were transferred to the laboratory of Aquaculture at the Department of Veterinary Medical Sciences of the University of Bologna for hemolymph collection (Cesenatico). The twelve animals excluded from the analysis were females and were released at the capture site. Only male crabs were used to standardize results and minimize sex-related variability. As molting in males of this species can occur at multiple times throughout the year [23,24], and molt stage was not assessed for individual specimens, individuals at different molt stages may have been present within the sampled cohort. Crabs were anesthetized using 0.4 mL/L 2-phenoxyethanol to minimize stress during handling. Following anesthesia, the carapace width (distance between the lateral margins of the carapace) and body weight were recorded using a digital caliper (±0.01 mm precision) and a Radwag WLC balance (±0.1 g), respectively.

### 2.2. Hemolymph Collection and Processing

Hemolymph was withdrawn from the pericardial sinus by inserting a pre-chilled (to minimize coagulation) 3 mL syringe fitted with a 23G needle through the arthrodial membrane at the dorsal base of the fifth pereiopod (swimming leg) [25], without any antiseptic preparation. At least 1 mL of hemolymph was collected per crab, avoiding contamination from other tissues. Samples were transferred into 2.6 mL tubes with serum gel clot activator. Preliminary trials indicated that tubes with citrate (0.106 mol/L, 3.0 mL) were unsuitable, as citrate chelates calcium and it could interfere with analyses. Freshly collected hemolymph samples were maintained at room temperature for approximately 20 min to allow clot formation, followed by centrifugation for 20 min at 3000× *g* and 20 °C, avoiding thermal fluctuations. After the first centrifugation, the clot was gently detached from the tube wall using disposable sticks, and samples were centrifuged again at the same g. This procedure was repeated 2–3 times until the supernatant was clearly separated from the clot. The supernatant was then transferred into 1.5 mL Eppendorf tubes and stored at −80 °C until subsequent biochemical analyses.

### 2.3. Biochemical Analysis

The hemolymph supernatant samples were analyzed at the Clinical Pathology service of the Department of Veterinary Medical Sciences of the University of Bologna. All samples were processed using an automated analyzer (AU480; Beckman Coulter, Brea, CA, USA). The following analytes were measured: total protein (photometric colorimetric test, Total Protein OSR6632 kit); aspartate transaminase (AST) (kinetic UV test, AST OSR6109 kit); total magnesium (photometric colorimetric test, Magnesium OSR6189 kit); total calcium (photometric colorimetric test, Calcium Arsenazo OSR6117 kit); phosphate (photometric UV test, Inorganic Phosphorus OSR6122 kit); sodium, potassium, and chloride (indirect quantitative determination, ISE Low Serum Standard 66317 and ISE High Serum Standard 66316); glucose (enzymatic UV test, Glucose OSR6121 kit); urea (kinetic UV test, Urea/Urea Nitrogen OSR6134 kit); uric acid (enzymatic colorimetric test, Uric Acid OSR6198 kit); triglycerides (enzymatic method, Triglyceride OSR6118 kit); and gamma-glutamyl transferase (GGT) (kinetic colorimetric test, GGT OSR6120 kit) (all reagents from Beckman Coulter, Brea, CA, USA).

The leftover hemolymph samples were used for internal validation. To evaluate analytical accuracy, recovery and linearity tests were performed. Baseline concentrations for glucose, total calcium, magnesium, triglycerides, urea, uric acid, AST, phosphate, total protein, sodium, potassium, and chloride were initially determined for two distinct hemolymph samples. A 1:1 pool was then prepared by combining equal volumes (900 μL each) of both samples. The expected values for the resulting pool were calculated as the arithmetic mean of the two starting concentrations. This pool was then analyzed in triplicate for the aforementioned variables to determine the recovery percentage. To evaluate the analytical linearity, a pool of two *C. sapidus* hemolymph leftover samples was diluted with distilled water to obtain seven concentration levels (0%, 6.25%, 12.25%, 25%, 50%, 75%, and 100%). Each dilution was analyzed in triplicate to evaluate total calcium, magnesium, total protein, sodium, potassium, and chloride, and the linearity of each analyte was assessed. To calculate the biochemical stability of *C. sapidus* hemolymph after freezing and prolonged conservation, a subset of leftover samples (*n* = 9) was subjected to a freeze–thaw cycle. Baseline concentrations of glucose, total calcium, magnesium, triglycerides, urea, uric acid, AST, phosphate, total protein, sodium, potassium, and chloride were determined immediately after collection on fresh samples. Subsequently, the samples were stored at −80 °C. After a storage period of six months, the samples were thawed at room temperature, re-analyzed and the results were compared with those obtained in fresh samples.

### 2.4. Statistical Analysis

Outliers were identified and removed using the Median Absolute Deviation (MAD) method with a threshold of k = 3. Descriptive statistics for each analyte are reported as mean ± standard deviation (SD) for normally distributed variables and median (min–max) for non-normally distributed variables. Data normality was assessed graphically and by using the Shapiro–Wilk test. For each variable, the 5th and 95th percentiles (p05, p95) were also calculated and reported as descriptive measures of the observed distribution. A *p*-value < 0.05 in the Shapiro–Wilk test was considered indicative of a significant deviation from normality. All analyses were performed using RStudio (latest version 2026.05.1, R Foundation for Statistical Computing, Vienna, Austria). Statistical analyses of the internal validation data were performed using Jamovi Cloud (version 2.7.24.0; Sydney, Australia). Analytical accuracy (recovery) was calculated as the percentage ratio between the mean measured value and the theoretical expected value, and results were compared against an acceptability range of 80–120%. Linearity was evaluated using linear regression analysis and considered optimal if R^2^ > 0.90. Post-storage stability was assessed by comparing baseline and post-thaw values using the Wilcoxon signed-rank test for paired data, as a non-parametric statistic was preferred due to the small sample size (*n* = 9) and a *p*-value > 0.05 was considered indicative of maintained biochemical integrity.

## 3. Results

Body weight of the collected crabs was 241.6 ± 72.36 g, and carapace width was 14.92 ± 1.73 cm. Over the sampling period, water temperature was 25.95 ± 3.47 °C, salinity 31.15 ± 1.48, and dissolved oxygen 7.7 ± 0.70 mg/L.

**Figure 1:**
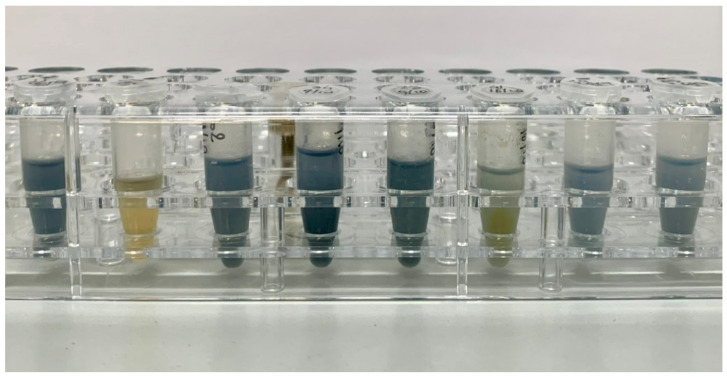
Examples of *C. sapidus* hemolymph macroscopic aspect after centrifugation.

After centrifugation, the hemolymph color was macroscopically evaluated, and the pigmentation identified among individuals ranged from pale yellow/green to light blue; the pigmentation was moderate, with no turbidity present after centrifugation (Figure 1).

To evaluate the analytical performance of the chemistry biochemical assays on *Callinectes sapidus* hemolymph, recovery, stability and linearity tests were performed.

The recovery test was conducted to evaluate the potential sample effect by mixing two hemolymph samples with known concentrations: Sample A and Sample B. The expected values were mathematically calculated based on an equal mixture of both samples (A + B) and compared against the measured values, which were calculated as the mean of three analytical replicates (Table 1a). The recovery percentages for most analytes fell within an acceptable analytical range (from 97.04% to 108.33%). Specifically, urea demonstrated an optimal recovery of 100.00%, while uric acid showed the lowest recovery at 97.04%. Conversely, AST exhibited a marked overestimation in the mixed sample, with a recovery rate of 150.00% (Table 1a).

Regarding sample stability after long-term storage at −80 °C (Table 1b), the Wilcoxon signed-rank test revealed that several analytes remained stable, showing no statistically significant differences from baseline values. These included urea (*p* = 0.074), uric acid (*p* = 0.570), calcium (*p* = 0.834), phosphate (*p* = 0.250), magnesium (*p* = 1.000), triglyceride (*p* = 0.057), and total protein concentration (*p* = 0.910). In contrast, a significant decrease in post-storage was observed for AST activity, which dropped from a baseline median of 11.00 U/L to 5.00 U/L (Relative Change: −86.00%, *p* = 0.014). Furthermore, electrolytes showed a mild but statistically significant increase after the freeze–thaw cycle: sodium increased by +4.85% (*p* = 0.009), potassium by +4.74% (*p* = 0.009), and chloride by +6.06% (*p* = 0.009). Glucose also showed a significant increase of +11.93% (*p* = 0.022).

**Figure 2:**
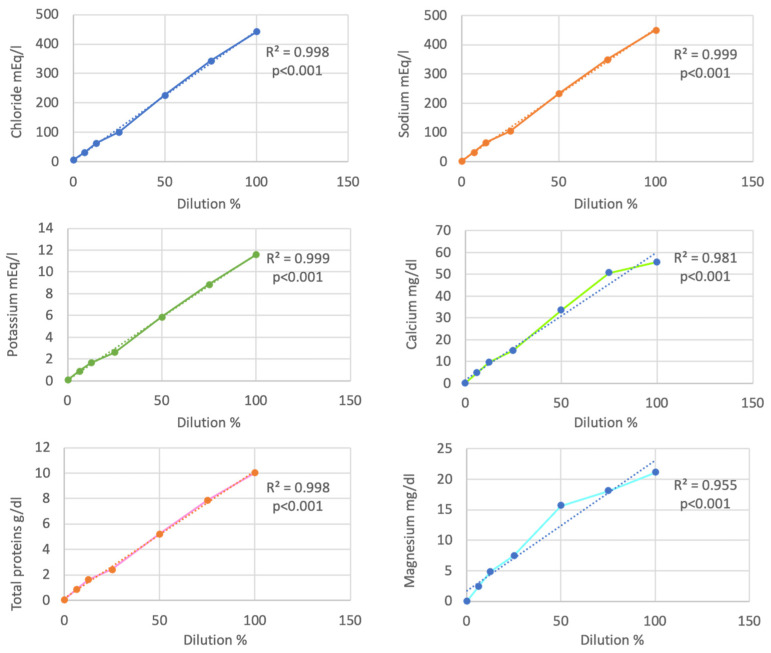
Analytical linearity plots for chloride, sodium, potassium, calcium, total proteins and magnesium analysis on *C. sapidus* hemolymph samples. The solid line with filled circles represents the measured concentrations at the seven different dilutions (0%, 6.25%, 12.25%, 25%, 50%, 75%, and 100%), derived from the mean of the three replicates, while the dashed line represents theoretical ideal linearity.

The linearity of the analyses was evaluated across seven serial dilutions (0%, 6.25%, 12.25%, 25%, 50%, 75%, and 100%) for chloride, sodium, potassium, calcium, total proteins, and magnesium. All tested analytes showed an excellent linear correlation, with coefficients of determination (R^2^) ranging from 0.955 for magnesium to 0.999 for sodium and potassium (*p* < 0.001 for all analytes; Figure 2).

Table 2 reports the values of hemolymph biochemical analytes after statistical analysis. Details of all excluded individuals, including missing or outlier data for each analyte, as well as sample availability and data-quality exclusions across analytes and samples are provided in Supplementary Table S1 and Figure S1.

**Figure 3:**
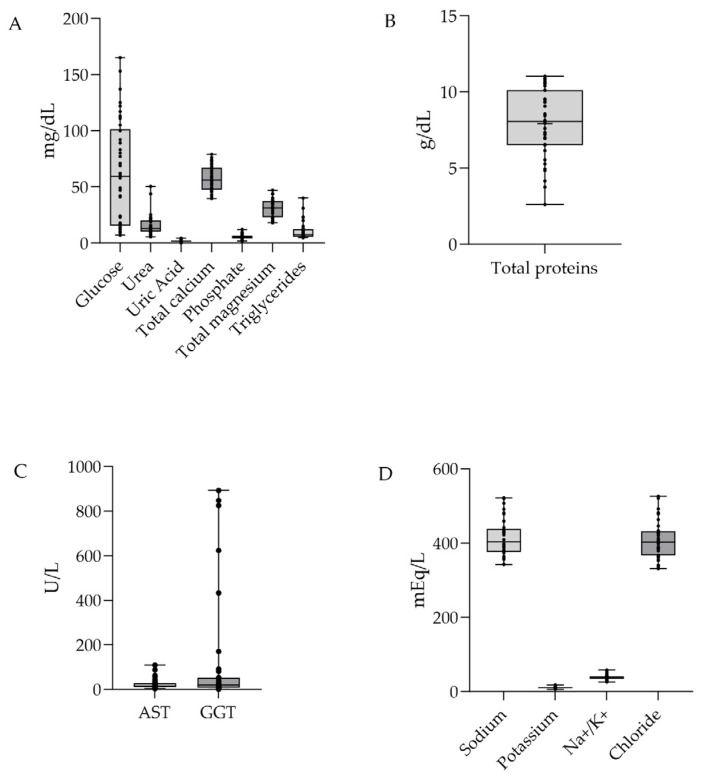
Visual distribution of hemolymph biochemical parameters measured in *C. sapidus*: (**A**) Glucose, Urea, Uric acid, Total calcium, Phosphate, Total magnesium, Triglycerides (mg/dL), (**B**) Total proteins (g/dL), (**C**) AST, GGT (U/L), (**D**) Sodium, Potassium, Na^+^/K^+^ and Chloride (mEq/L). Separate panels were used due to differences in units of measurement. Box plots represent the interquartile range (IQR; 25th–75th percentile), horizontal lines indicate the median and dots represent individual values.

The visual distribution of biochemical parameters is illustrated in Figure 3. Glucose showed high variability and a right-skewed distribution, with several values exceeding the 95th percentile: in contrast, phosphate and triglycerides displayed moderate variability, whereas urea, uric acid, total calcium and total magnesium showed comparatively lower variability and a more compact distribution (Figure 3A). For total proteins (Figure 3B), eight out of fourteen analytes deviated significantly from normality (Shapiro–Wilk *p* < 0.05), as detailed in Table 2. GGT is markedly variable (Figure 3C), which displayed extreme outliers reaching approximately 850 U/L, consistent with its significant non-normal distribution (Shapiro–Wilk *p* < 0.001). Sodium and chloride (Figure 3D) showed compact distributions centered around 400 mEq/L, while potassium and Na^+^/K^+^ remained considerably lower.

## 4. Discussion

In this study, a batch of male blue crabs was sampled, and their hemolymph was collected to analyze various biochemical markers, including glucose, urea, uric acid, total calcium, phosphate, magnesium, and triglycerides (mg/dL), AST, GGT (U/L), sodium, potassium (mEq/L) and total proteins (g/dL). Given the lack of specific studies on treating *C. sapidus* hemolymph, which tends to coagulate and whose blue color due to hemocyanin can interfere with analysis, this research contributed to develop a reliable protocol for hemolymph handling, while also providing biochemical data on the physiological condition of field-collected individuals, reflecting the natural variability associated with multiple interacting factors.

The main critical issue encountered in the handling of *C. sapidus* hemolymph samples was its marked tendency to undergo rapid coagulation immediately after collection, a phenomenon well documented in crustaceans and associated with the prompt activation of hemolymphatic defense mechanisms [26]. Preliminary attempts to use citrate-containing tubes as anticoagulants were unsuitable, likely due to calcium chelation, which may interfere with biochemical analysis. The use of serum gel tubes with clot activator, multiple centrifugation cycles and manual detachment of the clot from the tube wall made sample processing more labor-intensive than standard serum centrifugation in mammals.

An additional critical aspect observed during the study was the variability in hemolymph color which ranged from pale yellow/green to light blue among individuals. Unlike vertebrates, hemocyanin, the main oxygen-carrying pigment in invertebrates [27], is dissolved in the hemolymph; consequently, exposure to air causes the hemolymph to develop a characteristic bluish coloration [28]. Such variability raised concerns about potential interference with the colorimetric methods used in biochemical analyses. Preliminary tests with internal validation were therefore conducted to evaluate the impact of hemolymph color on analytical measurements. The AST data showed lower performance in both recovery and stability tests. These discrepancies may be primarily due to the baseline concentrations in *C. sapidus* hemolymph being extremely low and frequently close to the lower limit of detection of the chemistry analyzer. There was a mild increase in electrolyte concentrations (sodium, potassium, and chloride) and glucose following the six-month storage period, but this is likely attributable to a slight sample dehydration which may have occurred during processing or conservation, rather than an analytical error. Overall, these tests did not reveal significant analytical interference, suggesting that the adopted methodology is reliable enough regardless of the chromatic variability of samples.

### 4.1. Biochemical Composition

By exploring various biochemical parameters, this study aims to describe biochemical profile of a cohort of male *C. sapidus* collected in the northern Adriatic Sea during autumn, offering a preliminary but informative snapshot of their physiological condition under natural field conditions.

Regarding glucose content (62.34 ± 46.38 mg dL^−1^), levels were highly variable. Glucose plays a central role in the crustacean energy metabolism [29] and its mobilization is primarily regulated by the crustacean hyperglycemic hormone (CHH) [30]. Hemolymph glucose levels can vary throughout the molt cycle, which may represent one plausible source of variability: they tend to increase during intermolt and early premolt, reflecting the higher energy demand required for the synthesis of a new cuticle and preparatory processes for ecdysis, and decline during postmolt, as glucose is consumed to support body expansion, calcification, and exoskeleton hardening [31,32]. Thus, the cohort examined in the present study, exhibiting a wide range of hemolymph glucose concentrations from 7 to 165 mg dL^−1^, is consistent with a physiologically heterogeneous group of mixed-molt crabs with differing metabolic demands and energetic mobilization. However, the molt cycle likely represents only one source of variability. Other biological and environmental factors may also contribute to the observed differences, including nutritional status. Indeed, variations in hemolymph glucose are influenced by the quantity and quality of carbohydrates in the diet [33]. The high glucose values may reflect adequate nutritional status and sufficient dietary carbohydrate availability [34,35]. Dietary carbohydrates are stored as hepatopancreatic and muscle glycogen and are subsequently mobilized into the hemolymph under CHH control [34,36]. However, since hemolymph glucose in decapods is governed primarily by neuroendocrine (CHH-mediated) regulation rather than by direct dietary input, and the present design cannot isolate a dietary effect, an elevated glucose content may also indicate frequent activation of CHH axis, as physiological response also to acute stress [36]. Nevertheless, when compared to the values reported in the study on the Mediterranean green crab (*Carcinus aestuarii*) [37], the average glucose content is higher than the control group (37.8 ± 2.7 mg dL^−1^), but not comparable to stressed crab group (137.84 ± 16.2 mg dL^−1^), indicating that the crabs may not be under significant stress in the Adriatic conditions.

Hemolymph triglycerides in crustaceans serve as a readily accessible energy reserve [38], with circulating levels reflecting both nutritional status and molt stage (elevated during premolt, declining through ecdysis, and stabilizing during postmolt) [32,39,40]. Although no studies on *C. sapidus* report baseline hemolymph triglyceride levels under optimal conditions, the values recorded in the present study (7.84 ± 2.87 mg dL^−1^) fall within the baseline range reported for the control group of the Atlantic ghost crab, *Ocypode quadrata* (~12 ± 5 mg dL^−1^) [41], and are well below the levels reported for *C. sapidus* after seven days of forced fasting, which likely required mobilization of triglycerides to meet energy demands (~50–100 mg dL^−1^) [42]. This suggests a physiologically normal triglyceride profile, with no indication of metabolic alteration, further supporting the adequate nutritional condition of the examined cohort, previously inferred from hemolymph glucose levels. Since most crustaceans are predominantly ammoniotelic, urea and uric acid represent only minor end-products of nitrogen catabolism, with free amino acids serving as the primary intracellular osmolytes in response to salinity changes [43,44]. The low concentrations of urea (13.34 mg dL^−1^) and uric acid (1.51 mg dL^−1^) observed in the present study are therefore consistent with this excretory pattern. These metabolites, together with the enzymatic markers AST and GGT, reflect the broader picture of protein and amino acid catabolism. AST catalyzes the transamination of amino acids, most notably the aspartate–oxaloacetate interconversion, placing it at the intersection of amino acid catabolism and energy metabolism [45], while GGT is involved in glutathione metabolism and transmembrane amino acid transport [46]. Since urea and uric acid are the nitrogenous end-products of amino acid and purine catabolism respectively [47], their low circulating levels, combined with the low GGT activity, suggest a moderate and balanced protein turnover, with no evidence of acute tissue damage or significant metabolic stress. AST is reported for completeness but is excluded from physiological interpretation due to recovery failure observed during analytical validation. Although these analytes remain challenging to interpret in crustaceans, they have gained relevance as markers of cellular integrity and metabolic status in decapods [45,48] and their low activities in the present study further support the conclusion that the sampled cohort was in good overall physiological condition.

Calcium is the primary mineral responsible for hardening a crab’s new exoskeleton, with the hemolymph serving as both the transport medium and temporary reservoir for this ion throughout the molt cycle [49]. Research on *C. sapidus* and related crustaceans consistently documents pronounced molt-related shifts in hemolymph calcium and also magnesium concentrations, though most studies report trends or fold-changes rather than complete numerical datasets across all molt stages. Hemolymph Ca^2+^ has been shown to peak just prior to ecdysis, reflecting premolt calcium accumulation in preparation for carapace calcification, before falling to approximately half its peak value within one day postmolt, as calcium is rapidly mobilized toward mineralization of the newly formed exoskeleton [50]. Calcium and magnesium in crab hemolymph are closely interrelated in their physiological roles. Magnesium ions may modulate calcium uptake and exoskeleton formation by regulating the activity of alkaline phosphatase [51], while also acting as a natural calcium antagonist by controlling calcium channels and serving as a key cofactor for ATPases, including Ca^2+^-ATPase, thereby directly governing intracellular calcium influx and efflux [52]. This reciprocal relationship is also reflected across molt stages: hemolymph magnesium declines before ecdysis, likely to prevent premature calcification and to support muscle activity and tissue swelling, before rising sharply in the postmolt period and remaining elevated throughout rapid exoskeleton mineralization [53]. Given that, the specimens in the present study represented a mixed-molt cohort, the considerable variability observed in hemolymph calcium concentrations, ranging from 39.60 to 79.00 mg dL^−1^, and magnesium values, ranging from 18.08 to 47.00 mg dL^−1^, is consistent with the ionic profiles expected across individuals at different molt stages.

Inorganic phosphate, essential for energy transfer, as ATP and through phosphorylation reactions, cellular metabolism, and chitin synthesis, plays a central role in hemolymph mineral mobilization and is therefore tightly linked to the molt cycle [54]. During premolt, hemolymph phosphate rises, due to the increased ATP turnover and the mobilization of organic phosphate reserves to support muscle atrophy and new shell synthesis. Following ecdysis, phosphate levels drop before stabilizing at moderate concentrations [55]. The fluctuation of phosphate is connected to that of proteins through metabolic reallocation. The hemolymph is a protein rich fluid, containing hemocyanin, cryptocyanin (reservoir of building materials for new exoskeleton synthesis) and immune proteins as lectins and callinectin [7,56,57]. During premolt, as the old shell separates, muscle proteins are catabolized and released into the hemolymph alongside the liberated phosphate. While, in postmolt proteins are diluted since the crab absorbs large amounts of water to expand the new soft shell, as the phosphate withdrawn to harden the expanded exoskeleton [55]. However, hemolymph protein concentration and composition are also strongly influenced by the nutritional status of the crustacean. Under food-limited conditions, crabs may catabolize hemolymph proteins as an emergency energy source [58], while adequate dietary protein intake supports the synthesis of structural proteins and enzymes [7]. The phosphate values recorded in this study, ranging from 1.70 to 8.46 mg dL^−1^, likely reflect the mixed-molt composition of the sampled cohort, while the considerable variability in hemolymph protein concentrations, from 3.76 to 11.03 g dL^−1^, may additionally be influenced by the nutritional status of the individuals. It should be considered that, in general, the observed total protein content average value (8.10 ± 2.13 g dL^−1^) is greater than reported amounts of many native Mediterranean decapods. For example, hemolymph protein concentrations for shore crab *Carcinus aestuarii* typically range from 4.0 up to 6.0 g dL^−1^ [59]. Considering the hemolymph protein composition, its high content could be indicative of improved oxygen-carrying capacity, could reflect good nutrition condition and a general good health status of the examined cohort [60,61], matching the energetic demands of an aggressive, dynamic predator such as blue crab. According to Henry et al. (2012) [43], *C. sapidus* is known to have strong hyper-hypo-osmoregulatory capacity mediated by Na^+^/K^+^-ATPase in the posterior gills. The high concentrations of sodium (403.43 ± 41.78 mEq L^−1^) and chloride (401.75 ± 43.45 mEq L^−1^) in the crabs of the present study were measured under fully marine ambient salinity (~30–32), and these high ionic concentrations are consistent with effective ionic regulation under such conditions. These values are in close agreement with those reported for native Atlantic blue crab populations living in euryhaline environments and exceed those typical of several native Mediterranean decapods [12,43], suggesting that the sampled individuals were physiologically functional under the salinity conditions of the northern Adriatic. This physiological trait likely confers a competitive advantage in transitional coastal ecosystems characterized by high salinity variability [62].

### 4.2. Limitations

This study has several limitations. First, some analytes, such as glucose and electrolytes, showed statistically significant deviations after six months at −80 °C storage. However, these changes showed an average increase of approximately 5%, which is likely due to slight sample dehydration and such a limited shift is considered unlikely to affect the diagnostic interpretation of the chemistry profile in *C. sapidus*. Second, the analytical validation was restricted to a specific subset of analytes. For several other analytes, baseline hemolymph concentrations were close to the lower limit of detection of the clinical chemistry analyzer, precluding the assessment of linearity and recovery for those analytes. Consequently, while AST values are reported for comprehensiveness, they should be interpreted with caution as they likely reflect a physiological state where the enzyme is expressed at baseline levels below or at the threshold of chemistry analyzers. Third, although accuracy and linearity were evaluated, analytical precision was not determined. Since the accuracy results were within the acceptable range, we assumed that the analytical precision for these analytes was also reliable, as it is expected to align with the standard technical specifications of the automated analyzer used.

Furthermore, only a visual inspection of sample pigmentation was performed rather than a spectrophotometric quantification. Future studies establishing quantitative color grading could better clarify potential relationships between hemolymph color and other biochemical variables.

Additionally, in this study, the short-term stability at refrigerated temperature (4 °C) was not assessed; therefore, immediate processing or direct freezing of hemolymph samples remains recommended. However, to provide a practical timeline for field settings where immediate processing or freezing is unfeasible, data from mammalian sample processing may serve as a proxy. As established for mammalian samples, post-centrifugation specimens that are not analyzed immediately must be refrigerated for a maximum of 24–48 h, after which freezing is required [63].

Moreover, crabs were anaesthetized with 2-phenoxyethanol (0.4 mL/L) to minimize handling stress, a known trigger of CHH-mediated hyperglycemia. Since the anaesthetic was applied uniformly, any residual effect is systematic rather than a source of differential bias. Available evidence suggests it is well tolerated without altering key hemolymph analytes in related species [64,65]. However, its effects remain agent-, dose- and species-dependent, and no study has specifically validated this concentration for the analytes measured here. Future work pairing anaesthetized and unanaesthetized controls would allow direct quantification of this potential effect.

Finally, the study was carried out on thirty-eight male crabs from a single site over three months. This approach was suitable for the primary aim of developing and validating a standardized protocol for hemolymph collection and biochemical analysis but limits the generalizability of the reference values obtained. The observed physiological variability reflects the natural heterogeneity of a field-collected mixed-molt cohort yet cannot represent the full physiological range of the species across the northern Adriatic. Future studies should include multiple sites, seasons, and both sexes.

## 5. Conclusions

The standardization of hemolymph sampling and analysis techniques is crucial for advancing research on the biology of *C. sapidus* and other marine species, particularly in the context of invasive species. Hemolymph is a vital physiological fluid in crustaceans, and its biochemical composition reflects key indicators of health, stress, and metabolic status. The implementation of a standardized protocol for hemolymph chemistry analysis, supported by an internal validation test, ensures that these measurements are consistent and reproducible, confirming the method’s reliability despite hemolymph chromatic variability. Additionally, the hemolymph biochemistry of the sampled blue crabs indicates a potentially good physiological health status, although biochemical profiles showed considerable variability among individuals, as expected in field-collected samples, providing biochemical data on key hemolymph components useful for monitoring the species’ physiological and health status.

## Figures and Tables

**Table 1:** (**a**). Recovery test data for *C. sapidus* hemolymph samples. Expected values represent the theoretical concentration of the mixed samples, while the means represent the mean of three analytical replicates. Recovery % indicates the ratio between measured and expected concentrations. (**b**). Stability of biochemical variables in *C. sapidus* hemolymph samples after storage at −80 °C (*n* = 9). Values are expressed as median (Min–Max), mean difference and relative change. Baseline values represent fresh samples, while post-storage values represent samples analyzed after the freeze–thaw cycle.

**(a)**					
**Analyte**	**Sample A Measured Value**	**Sample B Measured Value**	**Expected Value** **(A + B)**	**Measured Value** **(Mean of 3 Replicates)**	**Recovery %**
Glucose (mg/dL)	75.00	28.00	51.50	55.33	107.44
Calcium (mg/dL)	64.50	52.20	58.35	61.23	104.94
Magnesium (mg/dL)	33.17	24.86	29.02	30.84	106.30
Triglyceride (mg/dL)	14.00	10.00	12.00	13.00	108.33
Urea (mg/dL)	19.19	15.73	17.46	17.46	100.00
Uric acid (mg/dL)	1.93	1.56	1.75	1.69	97.04
AST (U/L)	10.00	10.00	10.00	15.00	150.00
Phosphate (mg/dL)	5.55	5.46	5.51	5.93	107.72
Total Protein (g/dL)	7.80	9.75	8.78	9.01	102.66
Potassium (mEq/L)	11.60	11.2	11.40	11.53	101.17
Sodium (mEq/L)	455.00	424.00	439.50	448.67	102.09
Chloride (mEq/L)	451.00	424.00	437.50	442.00	101.03
**(b)**					
**Analyte**	**Baseline Value**	**Post Storage Value**	**Mean Difference** **(Wilcoxon Signed-Rank)**	**Relative Change %**	***p*-Wilcoxon** **Signed-Rank**
Glucose (mg/dL)	42.00 (10.00–100.00)	42.00 (17.00–102.00)	5.01	+11.93	0.022
Urea (mg/dL)	12.3 (9.26–50.30)	16.00 (9.87–49.10)	1.91	+15.52	0.074
Uric acid (mg/dL)	1.88 (0.78–2.56)	2.03 (0.88–8.84)	0.08	+4.52	0.570
AST (U/L)	11.00 (5.00–56.00)	5.00 (3.00–29.00)	9.46	−86.00	0.014
Calcium (mg/dL)	58.60 (46.50–73.00)	60.80 (49.50–72.80)	0.49	+1.60	0.834
Phosphate (mg/dL)	4.75 (3.26–9.40)	4.67 (3.79–10.90)	0.36	+7.58	0.250
Magnesium (mg/dL)	31.70 (20.90–40.20)	30.10 (25.80–38.00)	−0.35	−1.10	1.000
Triglyceride (mg/dL)	6.00 (5.00–40.00)	7.00 (5.00–60.00)	2.00	+33.33	0.057
Total Protein (g/dL)	9.07 (4.84–10.60)	9.56 (4.74–11.40)	−0.025	−0.27	0.910
Sodium (mEq/L)	392.00 (343.00–435.00)	424.00 (371.00–447.00)	19.00	+4.85	0.009
Potassium (mEq/L)	9.50 (6.40–12.00)	10.50 (6.60–12.30)	0.45	+4.74	0.009
Chloride (mEq/L)	388.00 (340.00–426.00)	416.00 (366.00–442.00)	23.50	+6.06	0.009

**Table 2:** Descriptive statistics of hemolymph biochemical analytes after outlier removal (MAD, k = 3).

Analyte	*N*	Mean ± SD	Median ± IQR	Min	Max	p05	p95	*p* Shapiro-Wilk
Glucose (mg/dL)	38	62.34 ± 46.38	59.50 ± 77.25	7.00	165.00	9.95	137.80	0.009
Urea (mg/dL)	34	13.34 ± 4.68	12.38 ± 4.80	4.68	22.23	6.61	21.12	0.035
Uric Acid (mg/dL)	33	1.51 ± 0.67	1.44 ± 1.02	0.08	3.09	0.64	2.63	0.808
AST (U/L)	29	15.41 ± 8.90	14.00 ± 11.55	3.00	37.00	5.00	31.00	0.051
GGT (U/L)	30	19.61 ± 18.67	12.15 ± 15.88	0.40	80.90	3.08	51.74	<0.001
Total Calcium (mg/dL)	33	57.56 ± 11.20	56.30 ± 18.50	39.60	79.00	40.86	74.60	0.042
Phosphate (mg/dL)	33	5.06 ± 1.64	4.80 ± 2.17	1.70	8.46	2.27	7.55	0.911
Total Magnesium (mg/dL)	32	31.23 ± 7.93	31.32 ± 13.72	18.08	47.00	18.82	43.87	0.470
Sodium (mEq/L)	35	403.43 ± 41.78	397.00 ± 57.00	342.00	491.00	342.00	478.60	<0.001
Potassium (mEq/L)	31	10.26 ± 1.56	9.90 ± 2.10	7.90	13.70	8.02	12.92	0.001
Na^+^/K^+^	34	36.38 ± 4.97	36.00 ± 6.25	26.00	47.00	26.00	43.25	<0.001
Chloride (mEq/L)	36	401.75 ± 43.45	399.50 ± 60.50	331.00	492.00	335.25	479.45	<0.001
Triglycerides (mg/dL)	25	7.84 ± 2.87	7.00 ± 4.50	5.00	15.00	5.00	12.70	0.057
Total proteins (g/dL)	34	8.10 ± 2.13	8.07 ± 3.14	3.76	11.03	4.07	10.86	0.118

## Data Availability

The original contributions presented in this study are included in the article/Supplementary Material. Further inquiries can be directed to the corresponding author.

## Supplementary Materials

The following supporting information can be downloaded at: https://www.mdpi.com/article/10.3390/ani16121894/s1, Table S1. Raw biochemical analytes’ values. Figure S1. Sample availability and data-quality exclusions across analytes and samples.

## Abbreviations

The following abbreviations are used in this manuscript:

AST	Aspartate transaminase
GGT	Gamma-glutamyl transferase
CHH	Crustacean hyperglycemic hormone
IQR	Interquartile range
