# Supplementary material for: Approach for Hemolymph Collection and Biochemical Profiling of Invasive Callinectes sapidus: Methodology and Physiological Assessment
Source: Animals (Basel). 2026 Jun 18;16(12):1894. doi: 10.3390/ani16121894 (PMC13296216; doi:10.3390/ani16121894)
Supplement: Supplementary file 1 [file animals-16-01894-s001.zip › animals-4345707-supplementary.pdf]

Supplementary information

Table S1. Raw biochemical analytes' values.

Raw values of the analytes for each specimens of *C. sapidus* analyzed. Cells highlighted in red color indicate values considered outliers. identified and removed using the MAD method with a threshold of k = 3. Cells highlighted in yellow color indicate missing values due to insufficient serum volume for processing.

Sample ID	Glucose mg/dL	Urea mg/dL	Uric acid mg/dL	AST U/L	GGT U/L	Total Calcium mg/dL	Phosphate mg/dL	Total Magnesium mg/dL	Sodium mEq/L	Potassium mEq/L	Na+/K+	Chloride mEq/L	Triglycerides mg/dL	Total proteins g/dL
1	71	14.44	1.72	27	9.0	79.0	4.41	34.5	442	8.1	55	421	7	8.13
2	89	50.34	2.10	56	8.0	64.0	5.89	31.7	435	10.5	41	409	9	10.57
3	13	20.34	1.16	21	24.4	58.4	5.17	31.4	436	8.7	50	426	7	10.90
4	49	20.37	1.44	32	23.3	73.0	4.55	40.2	422	8.5	50	406	7	10.53
5	100	10.32	1.22	15	20.4	66.0	4.75	32.4	373	6.4	58	353	8	7.24
6	18	9.26	2.00	6	11.0	56.3	4.27	37.8	392	9.5	41	403	5	7.35
7	92	13.20	0.89	11	623.8	59.7	8.46	36.3	429	11.1	39	433	23	6.15
8	42	9.94	2.14	5	5.6	58.6	4.20	30.9	430	12.0	36	426	5	4.84
9	62	11.04	2.11	16	52.4	41.4	4.77	31.1	378	9.8	39	379	10	5.28
10	113	11.26	1.65	8	432.9	71.0	7.29	36.9	432	9.2	47	421	23	8.07
11	111	7.23	0.99	3	36.4	46.7	2.70	31.2	425	10.5	40	427	5	
12	125	13.18	1.35	37	91.2	51.8	6.45	28.6	409	11.5	36	413	11	5.55
13	153	25.21	1.16		80.9		6.61		478	13.7	35	479	12	6.94
14	47	19.85	0.08		0.4				519	16.7	31	520		
15	165	43.92	0.92	109	893.1	73.0	12.10	44.0	459	17.7	26	446	31	10.84
16	58	20.29	1.88	9	848.0	68.0	9.40	38.6	364	10.0	36	361	40	7.21
17	122	18.92							507	17.6	29	492		
18	83	13.28	2.09	11	51.2	76.0	6.59	38.3	491	13.4	37	482	13	8.56
19	105	12.53	1.54	30	49.3	59.0	5.97	34.6	522	17.2	30	526	9	4.93
20	117	22.23	4.20	88	170.3		9.68		481	18.3	26	478	15	9.53
21	137	23.12	4.39	44	26.1	70.0	7.46	37.5	459	15.7	29	463	11	10.64

22	16	12.89	0.56	21	9.7	54.5	6.10	26.4	387	12.6	31	386	6	10.72
23	10	10.06	2.56	5	10.8	48.7	4.80	22.9	394	10.8	36	388	6	9.32
24	14	14.48	0.86	12	8.3	55.7	4.54	22.1	397	9.9	40	385	5	10.39
25	13	10.26	1.14	13	5.9	45.8	4.14	19.9	342	8.5	40	331		10.12
26	24	12.27	1.79	11	22.5	46.5	5.13	20.9	343	9.4	36	340	5	9.48
27	23	12.37	0.78	16	5.0	49.3	3.26	23.4	361	8.5	42	355	5	9.07
28	77	20.76	3.09	63	36.6	62.0	7.77	31.4	377	11.5	33	365	10	11.03
29	69	12.39	2.79	24	20.1	66.0	6.00	47.0	398	11.7	34	385	6	9.33
30	80	22.19	3.51	27	19.8	74.0	6.26		409	11.3	36	401		8.44
31	61	15.87	3.31	14	11.1		5.34	43.8	429	11.9	36	432	7	7.95
32	41	8.84	1.10		825.3	49.6	6.58	30.5	342	9.5	36	339	20	3.76
33	17	12.31	1.40	20	13.2	53.0	3.88	35.9	390	11.3	35	398	5	7.00
34	9	7.69	1.03	6	6.8	39.6	2.52	19.2	378	9.7	39	384		2.62
35	10	9.32	2.07	15	8.2	43.1	3.36	18.1	343	7.9	43	336		7.61
36	12	6.94	2.07	14	3.3	46.1	2.64	22.6	358	9.6	37	367	7	6.52
37	14	11.64	1.57	13	5.9	48.3	3.43		381	9.3	41	390		6.54
38	7	5.60	0.67	5	2.8	45.5	1.70	19.4	356	8.1	44	363		4.17
N. valid values	38	34	33	29	30	33	33	32	35	31	34	36	25	34
N. missing values	0	0	1	4	1	5	2	6	0	0	0	0	8	3
N. outliers values	0	4	4	5	7	0	3	0	3	7	4	2	5	1

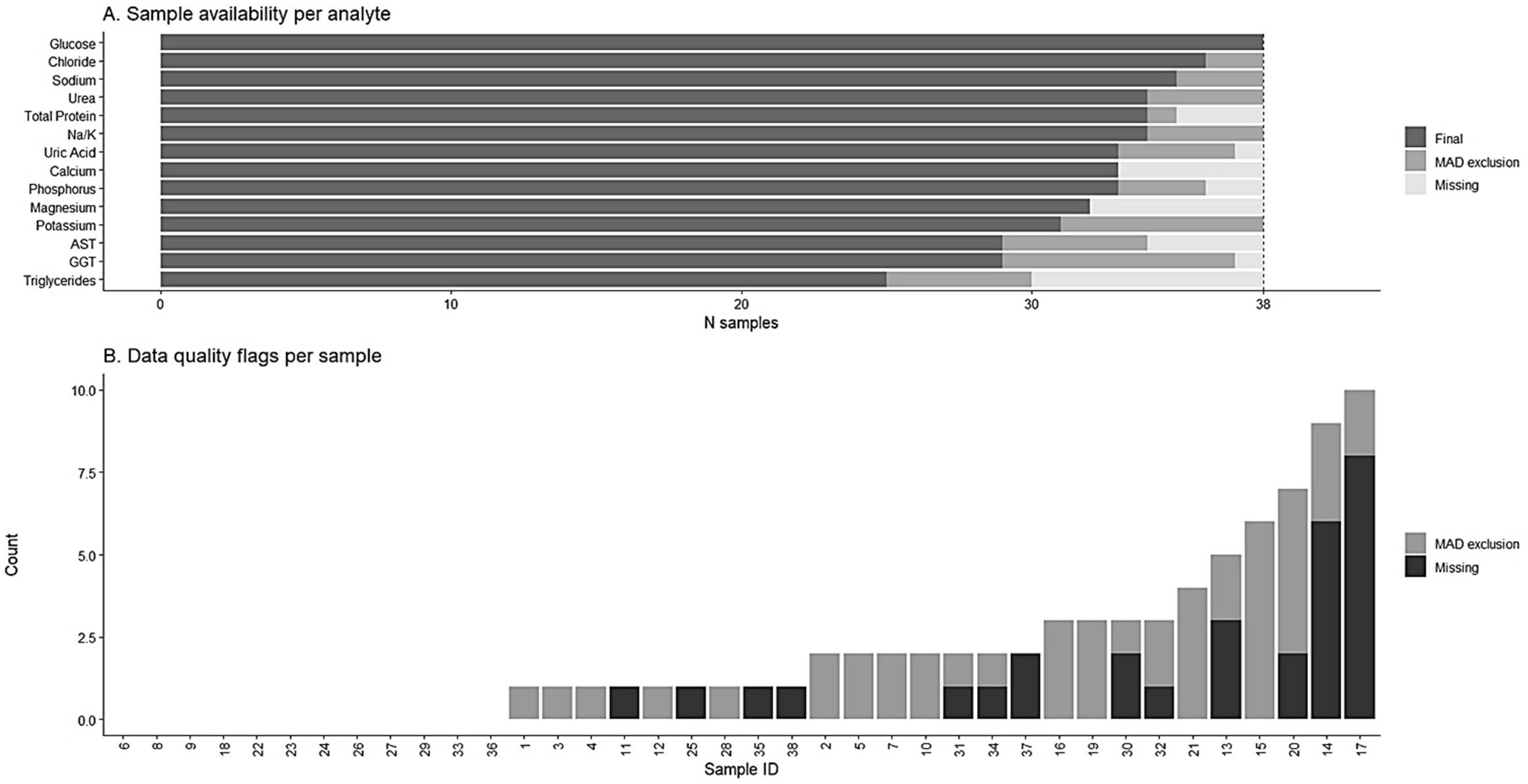

Figure S1. Sample availability and data-quality exclusions across analytes and samples.

(A) Stacked bar plot showing the number of valid observations retained for each analyte after quality control (dark grey), together with observations excluded as outliers based on the median absolute deviation (MAD) criterion (medium grey) and missing values (light grey). The dashed vertical line indicates the initial sample size (n = 38). (B) Number of missing values and MAD-based outlier exclusions for each individual sample across all analytes. Missing values were concentrated in a subset of individuals, likely reflecting insufficient serum volume available for all analytes, whereas MAD-based outlier exclusions were distributed across different individuals and analytes.
